# Supplementary material for: Combined Systemic Drug Treatment with Proton Therapy: Investigations on Patient-Derived Organoids
Source: Cancers (Basel). 2022 Aug 3;14(15):3781. doi: 10.3390/cancers14153781 (PMC9367296; doi:10.3390/cancers14153781)
Supplement: Supplementary file 1 [file cancers-14-03781-s001.zip › cancers-1814429-supplementary.pdf]

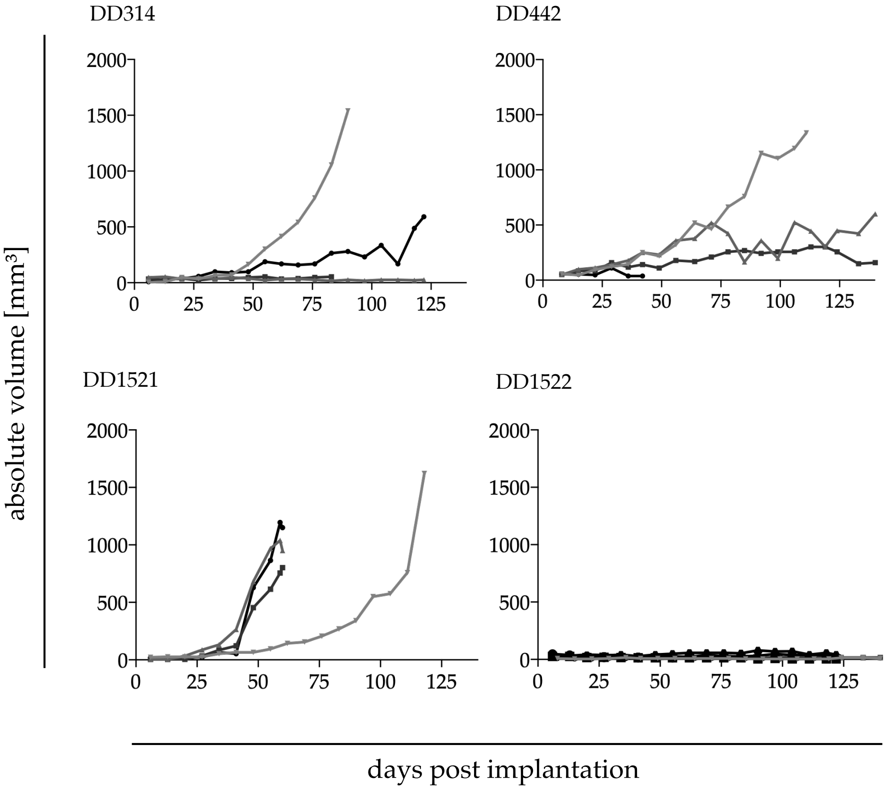

Figure S1: Tumor growth after subcutaneous implantation of PDOs in NMRI $^{nu/nu}$ mice. Growth of xenograft tumors were observed over 122 days for DD314, 140 days for DD442 and DD1522, 122 for DD1521. Two animals needed to be sacrificed due to bad general conditions (DD314, DD442).

Table S1: Number of implantetd confluent wells of an 48 well plate depending on attempt and PDO line

PDO	Attempt of implantation	Number of mice	Implanted wells per mouse
DD314	1st	4 in every experiment	3
DD442	1st		15
DD1521	1st		3
DD1522	1st		3
DD1522	2nd		12.5

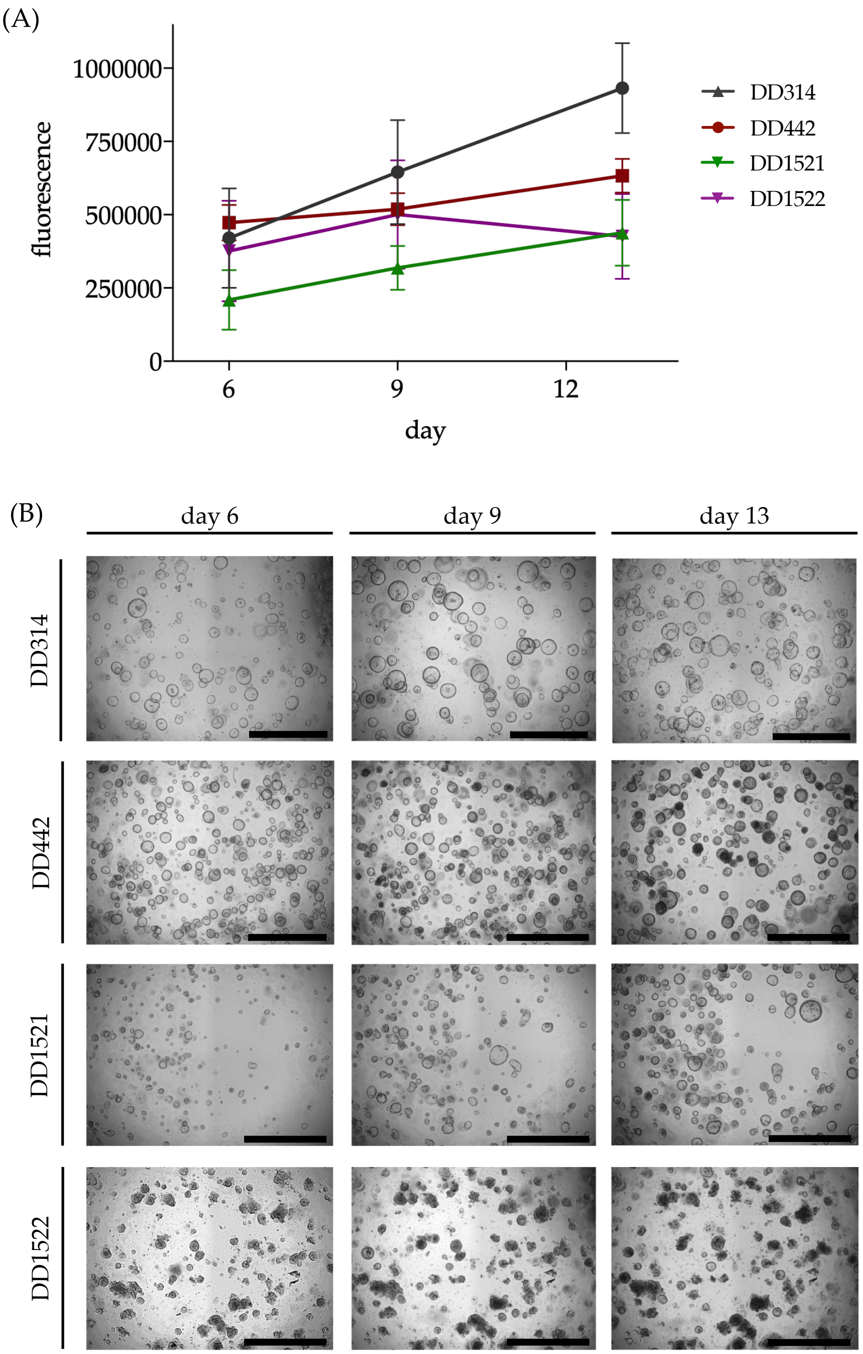

Figure S2: Proliferation of PDOs *in vitro* **(A)** Measured fluorescence of PrestoBlue™ Assay of untreated PDOs between d6 and d13 after seeding (n = 6). **(B)** Brightfield images showing untreated PDOs at different timepoints (scale bar: 500 µm).

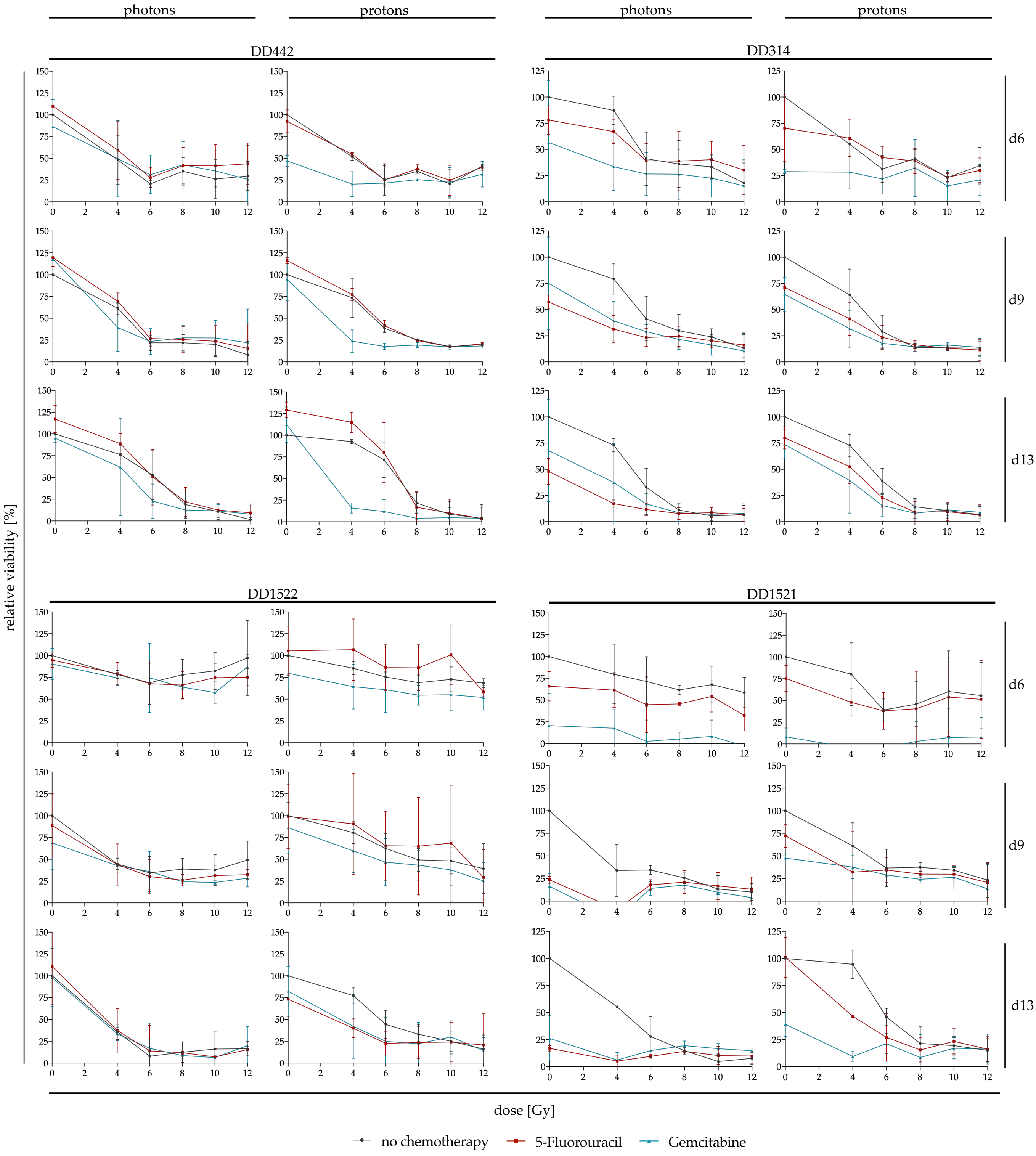

Figure S3: Summary of all combined radiotherapeutic treatments on PDOs. Relative viabilities were measured via PrestoBlue™ Assay (n = 2 for 4 Gy protons in DD314, DD442, DD1521 and photons in DD1521, all other conditions n = 3).

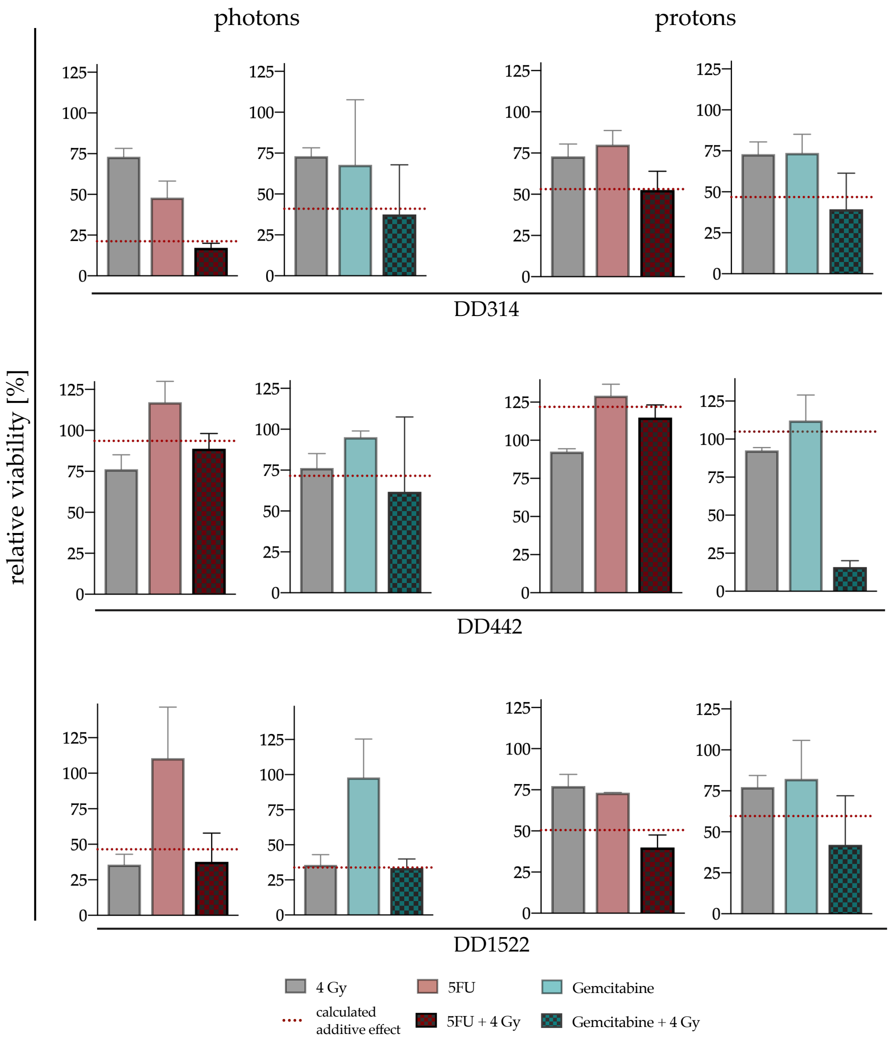

Figure S4. Response of PDOs to RCT with 4 Gy photons or protons. Relative viability was measured on day 13 after RCT with either gemcitabine or 5FU in combination with 4 Gy photon or proton irradiation, respectively ± SD. The red dotted line shows the sum of effects after single treatments (n = 2 for protons in DD314 and DD442, all other conditions n = 3).
